# Mobile Apps for Dental Caries Prevention: Systematic Search and Quality Evaluation

**DOI:** 10.2196/19958

**Published:** 2021-01-13

**Authors:** Rebecca Chen, Karla Santo, Grace Wong, Woosung Sohn, Heiko Spallek, Clara Chow, Michelle Irving

**Affiliations:** 1 Sydney School of Dentistry Faculty of Medicine and Health The University of Sydney Westmead Australia; 2 Westmead Applied Research Centre Faculty of Medicine and Health The University of Sydney Westmead Australia; 3 Northern Sydney LHD, NSW Health Sydney Australia

**Keywords:** dental caries, oral hygiene, self-management, mobile applications

## Abstract

**Background:**

Dental caries is the most common multifactorial oral disease; it affects 60% to 90% of the global population. Dental caries is highly preventable through prevention behaviors aimed at improving oral hygiene, adequate fluoride usage, and dietary intake. Mobile apps have the potential to support patients with dental caries; however, little is known about the availability, target audience, quality, and features of these apps.

**Objective:**

This review aims to systematically examine dental caries prevention apps; to describe their content, availability, target audience, and features; and to assess their quality.

**Methods:**

We systematically identified and evaluated apps in a process paralleling a systematic review. This included a search strategy using search terms; an eligibility assessment using inclusion and exclusion criteria focused on accessibility and dental caries self-management behaviors, including oral hygiene, dietary intake, and fluoride usage; data extraction on app characteristics, including app store metrics; prevention behavior categorization; feature identification and description; a quality appraisal of all apps using the validated Mobile App Rating Scale (MARS) assessment tool; and data comparison and analysis.

**Results:**

Using our search strategy, we retrieved 562 apps from the Google Play Store and iTunes available in Australia. Of these, 7.1% (40/562) of the apps fit our eligibility criteria, of which 55% (22/40) targeted adults, 93% (37/40) were free to download, and 65% (26/40) were recently updated. Oral hygiene was the most common dental caries prevention behavior domain, addressed in 93% (37/40) of the apps, while dietary intake was addressed in 45% (18/40) of the apps and fluoride usage was addressed in 42% (17/40) of the apps. Overall, 50% (20/40) of the apps addressed only 1 behavior, and 38% (15/40) of the apps addressed all 3 behaviors. The mean MARS score was 2.9 (SD 0.7; range 1.8-4.4), with 45% (18/40) of the apps categorized as high quality, with a rating above 3.0 out of 5.0. We identified 21 distinctive features across all dental caries prevention behaviors; however, the top 5 most common features focused on oral hygiene. The highest-ranking app was the *Brush DJ* app, with an overall MARS score of 4.4 and with the highest number of features (n=13). We did not find any apps that adequately addressed dental caries prevention behaviors in very young children.

**Conclusions:**

Apps addressing dental caries prevention commonly focus on oral hygiene and target young adults; however, many are not of high quality. These apps use a range of features to support consumer engagement, and some of these features may be helpful for specific patient populations. However, it remains unclear how effective these apps are in improving dental caries outcomes, and further evaluation is required before they are widely recommended.

## Introduction

### Background

Dental caries is a preventable, noncommunicable multifactorial disease that affects 60% to 90% of the population globally [1,2]. When left untreated, end-stage management of dental caries can result in pain, infection, and facial swelling, leading to emergency department presentations, especially for young children [3,4]. Although highly preventable, dental caries resulted in 70,200 preventable hospitalizations in Australia from 2016 to 2017 [5]. Dental caries prevention can be achieved at the individual level by addressing specific prevention behaviors, including adequate oral hygiene practices [3], age-appropriate topical fluoride usage [6], and diet modifications that reduce the amount of free sugar consumption [7]. At present, prevention behavior change interventions have included patient-focused dietary counseling and oral hygiene instruction, mostly delivered alongside operative clinical interventions in clinical settings [8,9]. However, these interventions are time intensive, workforce intensive, and expensive to deliver; also, without regular and repeated exposure, these interventions have shown inconsistent results on sustainably improved dental caries outcomes [9,10].

Growing mobile phone ownership globally and integration with the internet [11,12] have prompted the development of and research into mobile health (mHealth) interventions to address a broad range of behavior change practices for chronic disease management. These mHealth tools seek to modify a range of broad and specific behavioral factors related to diet [13], exercise [14], and medication adherence [15,16] to manage a range of chronic conditions, including diabetes [17,18], obesity [19,20], and cardiovascular diseases [15]. A variety of mHealth interventions have shown promising results in a variety of populations across the lifespan [21,22] and have particularly provided equitable support to remote, regional, and underserved populations [23-27]. Thus, it is necessary to both use and assess mHealth as a viable modality to support behavior change in the management of a range of noncommunicable diseases, including oral diseases, namely dental caries.

### Apps for Dental Caries Prevention

Dental caries has many modifiable risk factors common to other noncommunicable diseases [4,28], driving a rationale for the adoption of innovative disease management approaches, including mHealth. Current research in mHealth for oral health has largely focused on addressing periodontal diseases through motivation for oral hygiene, with the delivery of simple text messages [29-31]. A recently published systematic review of the literature, focused on oral hygiene alone, highlighted the potential of mHealth interventions to improve oral health knowledge alongside modest clinical improvements in gum health in the adult and adolescent populations [29]. However, it remains unclear whether these results can be extended to address other dental caries risk factors, including a cariogenic diet and inadequate fluoride usage. Although important across the lifespan, preventive behaviors associated with appropriate fluoride usage and low sugar diets, including the timing of consumption are particularly influential in decreasing dental caries risk during the unique developmental stages of children aged younger than 6 years [3,8]. Uninformed parents could be at greater risk of their child experiencing a preventable hospitalization because of dental caries [5,10]. Although previous studies have focused on oral hygiene [29,32], it is important for this study to systematically scope the target audience and range of apps that addressed other dental caries prevention behaviors, including adequate fluoride usage and dietary modification.

Furthermore, 2 recent reviews on apps used in oral health focused on the information analysis of apps that targeted the adult population in the United States [33] and the United Kingdom [32]. These reviews found poor information quality and identified the need to comprehensively analyze the features available in the apps alongside the use of a validated quality rating scale. The Mobile App Rating Scale (MARS) is a validated scale that has been used in a wide range of health care contexts to comprehensively assess the quality of health apps. Further analysis of features also provides information on usability and the potential for longer-term engagement with an app. Therefore, this study aims to systematically examine oral health apps that address a range of modifiable dental caries prevention factors and to systematically describe their content, availability, target audience, features, and quality.

## Methods

### Systematic Search Strategy

**Figure 1:**
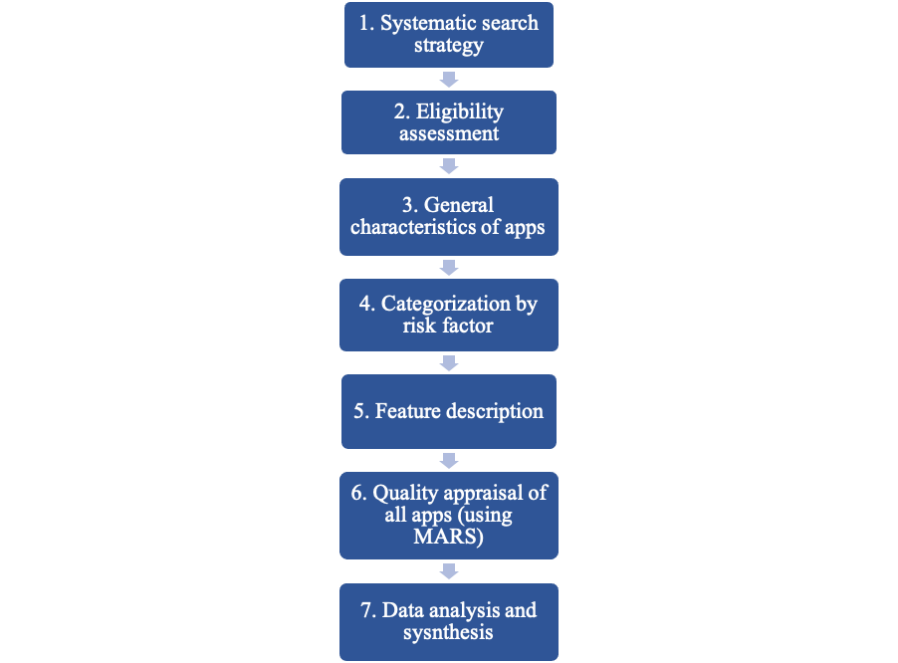
Schematic steps of the systematic review and quality evaluation. MARS: Mobile App Rating Scale.

This review was conducted using a stepwise approach according to a previously published methodology that parallels a systematic review (Figure 1) [15]. We searched the main app stores: Google Play Store and iTunes. Of the 5.6 million apps available internationally, Google Play offers 2.47 million apps, and iTunes offers 1.8 million apps [34]. The search was conducted on the Google Play Store and iTunes with the app store country and region set to Australia between January 8 and 19, 2019, using the top 8 key search terms. These search terms were chosen based on their performance in retrieving the highest number of relevant apps for dental caries prevention from preliminary searches. The final list was developed and agreed upon by all authors and focused on the self-management behaviors that support dental caries prevention across all age groups, including young children. These search terms included dental caries, early childhood caries, tooth decay, dental caries prevention, early childhood caries prevention, tooth decay prevention, saliva, and fluoride.

### Eligibility Assessment

All apps retrieved from the search were screened by 2 independent reviewers (RC and GW) for eligibility using prespecified inclusion and exclusion criteria (Textbox 1) that aimed to identify relevant apps that were accessible to most of the general public relevant to dental caries prevention.

**Inclusion and exclusion criteria.**

**Inclusion criteria**

- The app focused on supporting the general public of any age to address prevention behaviors associated with dental caries prevention
- The app addressed dental caries prevention factors: 
  Oral hygiene including toothbrushing
  Fluoride usage
  Diet modification
- The app was accessible to the care context of Australia.
- The app was in the English language.

**Exclusion criteria**

- Apps targeted at clinicians or student clinicians
- Apps that were not related to health and were considered arcade games only
- Apps that addressed other health or oral health issues but were not specific to dental caries
- Apps that were priced or had in-app purchases or electronic devices such as electric toothbrushes priced above Aus $3.00 (US $2.29), which is the average price that consumers are willing to pay for an app [35] to ensure affordability
- Apps that were associated with a specific health clinic

### General Characteristics of Apps

All apps that fit the inclusion criteria were downloaded on either the Android platform using a Samsung smartphone (Galaxy 9) or the iOS platform using an iPhone XR. The general characteristics of the apps were adapted from the *classification section* of the MARS tool (Multimedia Appendix 1) to describe the app name, app developer, date of the last update, platform (Google Play or iTunes), cost, star rating according to the app store, affiliations, target age groups, and focus of the app.

### Categorization by Prevention Factors

Apps were categorized according to broad modifiable factors associated with dental caries prevention: oral hygiene, fluoride, and diet. For apps to be classified as addressing oral hygiene, at minimum, information about the importance of good oral hygiene for dental caries prevention had to be present in the app. Additional information and features could include a video demonstration of tooth brushing or interdental cleaning techniques, such as dental flossing, or *gamification* of toothbrushing, defined as the use of game elements in nongame contexts [36]. For fluoride, some information about the effects of fluoride from various modalities, including toothpaste or water consumption related to dental caries prevention, could be included. For diet, information on specific diet changes that could influence dental caries outcomes, for example, the use of a traffic light grading system to educate and encourage users to swap food choices to alternatives with less sugar [37], could be included.

### Feature Description and Analysis

Features are elements of an app designed to increase interactivity and consumer engagement. For oral health apps, these features may include gamification and timers [33]. For the feature description, we identified and defined these through an iterative process combining terminology from previously published literature [15,38,39] with input from experts in the field and all authors. Further analysis was conducted to catalog the features of all apps stratified by the broad dental caries prevention factor each app addressed and to identify common features.

### Quality Appraisal of Apps

All apps that fit the inclusion criteria were evaluated for quality using the MARS (Multimedia Appendix 1). This scale provides a standardized approach with 19 objective items and provides appraisal across 4 subscales: engagement, functionality, esthetics, and information quality [38,40]. The engagement subscale appraises whether the app was fun, interesting, customizable, and interactive (eg, push notifications, sends alerts) to the target audience. The functionality subscale assesses whether the app is correctly functioning and easy to learn, with easy navigations and logical flow. The esthetic subscale provides appraisal with regard to the general visual appeal and stylistic consistency of the app. Finally, the information subscale assesses the quality of the information, for example, whether the textual information and references are from credible sources. The overall MARS also sets a minimum quality threshold score of 3.0 out of 5.0, providing the ability to identify *high-quality* apps to patients and clinicians or further analysis [41]. It has been used in various contexts with excellent internal consistency and interrater reliability [15,42-46]. In total, 2 independent reviewers (RC and GW) were trained to use the MARS instruments through a web-based training program created by the MARS developers [38]. Each reviewer independently spent at least 30 min to thoroughly test each app on both devices. Data on the objective subscales of the MARS and additional features of the apps were extracted and entered into an Excel (Microsoft Corporation) spreadsheet. The items were rated on a 5-point scale (1: inadequate, 2: poor, 3: acceptable, 4: good, and 5: excellent). Any disagreements between the 2 reviewers were resolved by taking a consensus discussion. We calculated the means of the MARS and interrater reliability scores between reviewers using SPSS version 22.0 (IBM Corporation). High-quality apps were determined from the overall threshold score of 3 out of 5 in the overall mean score as defined by the developers of MARS, providing the ability to identify *high-quality* apps for further analysis [38,41].

### Data Analysis and Synthesis

Further data analysis and synthesis were conducted based on the iteratively generated hypothesis from the initial MARS quality analysis. First, we wanted to compare the quality rating between apps that addressed a differing number and range of dental caries prevention factors. Second, the correlation between the MARS quality rating and the number of features across all apps was conducted.

## Results

### Systematic Search and Eligibility Assessment

**Figure 2:**
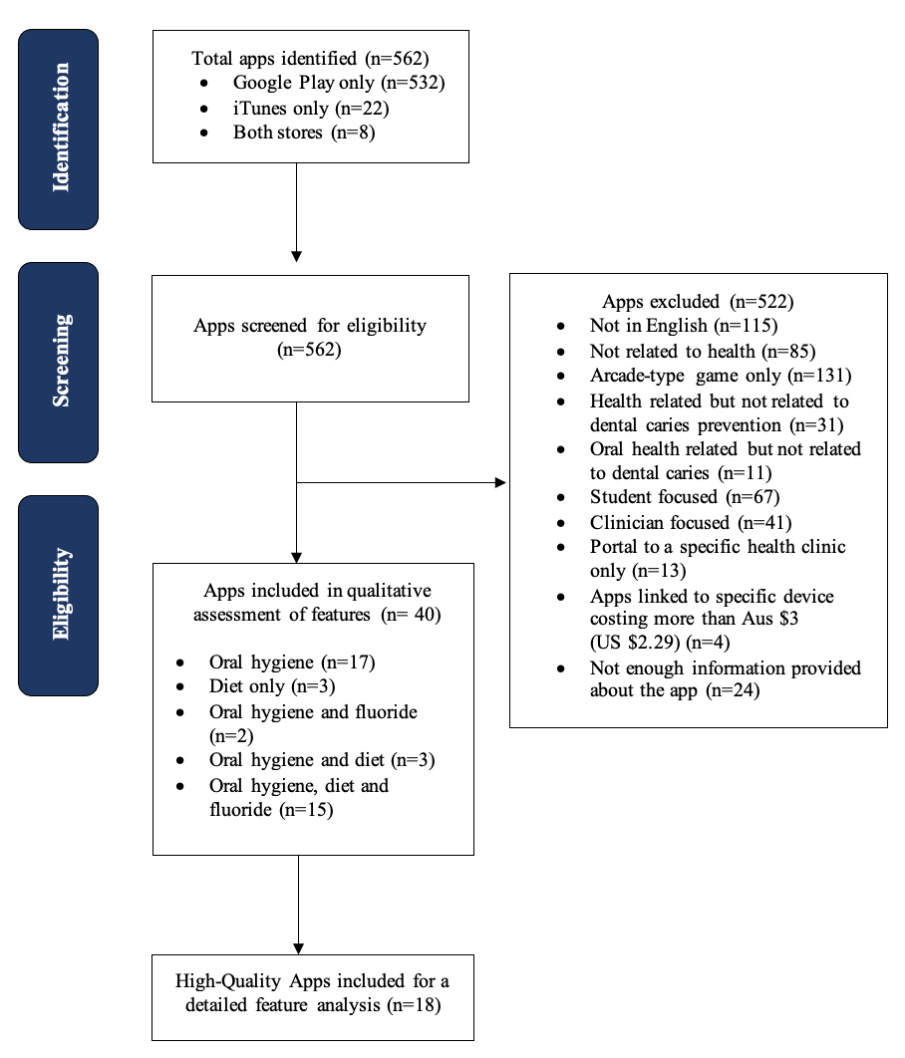
PRISMA (Preferred Reporting Items for Systematic Reviews and Meta-Analyses) flowchart of apps identified through the systematic search.

Our systematic search retrieved a total of 562 apps, with 532 (94.7%) apps identified in the Google Play Store alone, 22 (5.3%) apps identified in iTunes alone, and 8 (1.4%) apps found in both stores. These apps were screened for eligibility, and 92.9% (522/562) were excluded based on the inclusion or exclusion criteria. The reasons for exclusion are presented in Textbox 1. A total of 40 (7.1%) unique apps were included for further analysis (Figure 2).

### General Characteristics of Apps

Most of the apps (37/40, 93%) were free to download, with only 3 of the 40 apps (8%) incurring a cost between Aus $0.99 (US $0.75) and Aus $2.99 (US $2.29) on iTunes alone. More than half (26/40, 65%) of the apps included were recent and current, as they were last updated either in 2018 or 2019. A total of 29 out of 40 apps (73%) were available on the Google Play Store only, and 3 out of 40 apps (8%) were available on the iTunes store only; 20% (8/40) of the apps were available in both stores (Multimedia Appendix 2).

Most (27/40, 68%) of the apps’ affiliations were unknown, and 9 of 40 apps (23%) had clear commercial affiliations. Only 10% (4/40) of apps had affiliations with a university, and 50% (2/4) of these apps, namely, *Brush DJ* and *My Dental-Care - Your Guide to Oral Health*, had affiliations with the UK National Health Service, a government affiliation. More than half (22/40, 55%) of the apps were targeted toward adults or young adults, with 63% (14/22) of these apps also targeted adolescents. Apps classified as targeting a general audience were 18% (7/40), whereas 28% (11/40) of apps were targeted at children aged older than 7 years. When analyzing the focus of the app, half (20/40, 50%) of the apps focused on information provision, such as health-seeking behaviors. The other half of the apps provided additional behavior change prompts, with 70% (14/20) of the apps providing specific goal setting functions within the app.

### Categorization by Prevention Factor

**Figure 3:**
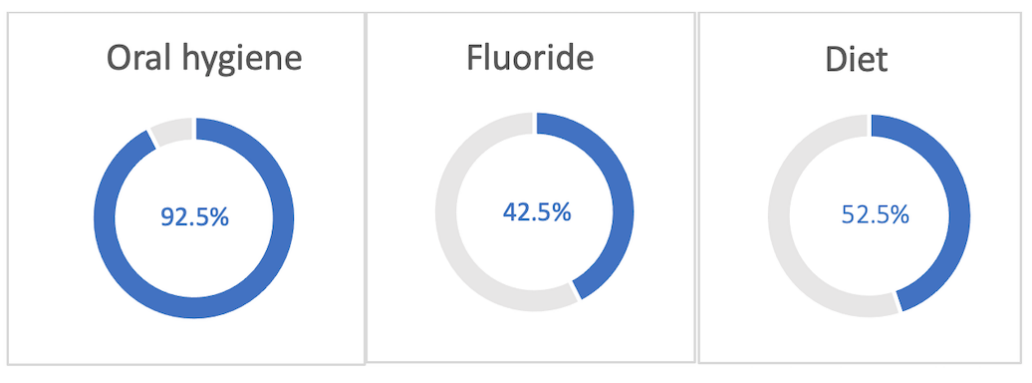
Percentage of all apps that addressed each prevention factor.

**Figure 4:**
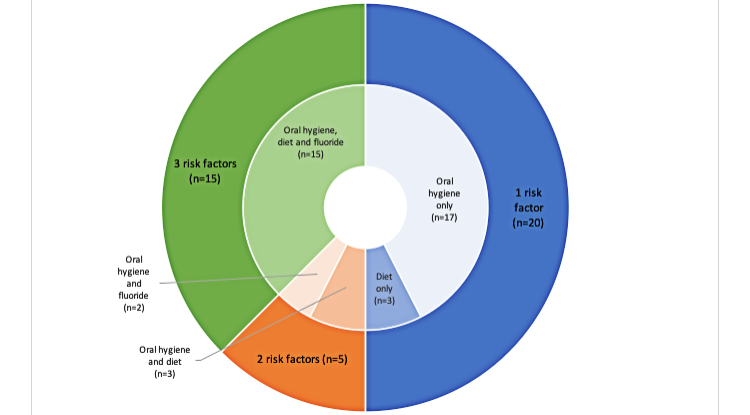
Categorization of apps according to the number and types of prevention factors addressed. (The outer rim indicates the number of prevention factors each app addressed. The inner rim shows the combination of the types of prevention factors that each app addressed).

Of the 40 included apps addressing a range of dental caries prevention factors, 37 (93%) addressed oral hygiene, 17 (43%) addressed fluoride, and 21 (53%) addressed diet (Figure 3). Furthermore, 50% (20/40) of apps addressed only one of these factors for oral health, 12% (5/40) of apps addressed 2 factors, and 38% (15/40) of apps addressed all 3 factors. Of the 20 apps that addressed only 1 factor, 17 (85%) addressed oral hygiene alone and 3 (15%) addressed diet alone. Of the 5 apps that addressed 2 factors, these combinations included oral hygiene and fluoride with 2 (40%) apps and oral hygiene and diet with 3 (60%) apps (Figure 4).

Of the 40 apps that fit our inclusion criteria, 37 (93%) addressed oral hygiene, 17 (43%) addressed fluoride, and 21 (53%) addressed diet.

### Feature Description and Analysis

**Figure 5:**
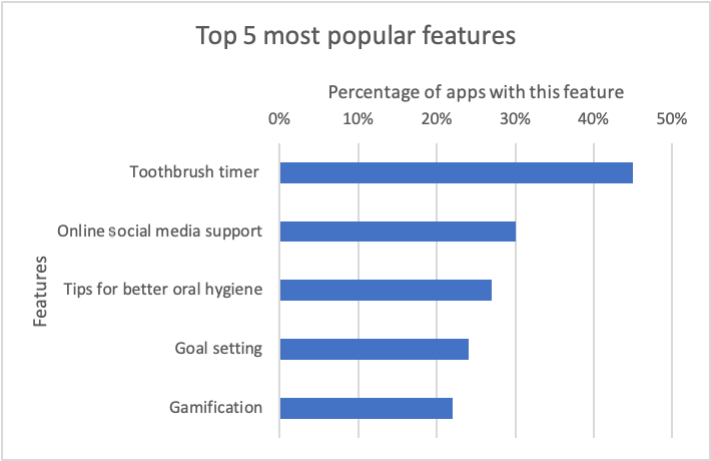
Top 5 most common features found in apps.

A total of 21 features were identified; these features were grouped into general and specific features related to each dental caries prevention factor, including oral hygiene, fluoride usage, and diet. The list of features and descriptions are outlined in Textbox 2; each feature was given a label to allow for a corresponding reference to Multimedia Appendix 3. Most of the unique features identified were associated with oral hygiene (9/21, 43% features), compared with fluoride (2/21, 10% of features) and diet (5/21, 24% of features; Multimedia Appendix 3). Other than web-based social media support, considered to be a general feature, the top 5 most common features found in all apps addressed oral hygiene (Figure 5). The *Brush DJ* app had the highest number of features at 13 (Multimedia Appendix 3). Of 37 of 40 apps that addressed oral hygiene, 17 (46%) had the *tooth brush timer* as the most common feature, followed by *goal setting* (9/37, 24% of apps), *gamification* (8/37, 22% of apps), and 5 apps (14%) with a video demonstration of oral hygiene techniques. The *Brush DJ* app and the *Disney Magic Timer* app had the highest number of oral hygiene–related features, with each of these apps having 78% (7/9) of these features. Only 12% (2/17) of apps that addressed the fluoride prevention factor adequately provided additional information and used visual aids to support the information provided, for example, the appropriate amount of fluoride toothpaste that should be dispensed for children. These 2 apps, namely, *My Dental-Care - Your Guide to Oral Health* and *Brush DJ*, were also the only apps that were affiliated with both a university and government health service. Of the 21 apps that addressed diet, the *Food For Teeth - Food* *Database and Diet Diary* app was the only app that included additional features such as an ability to record a diet diary, with a database of food items, including their pictures and serving size embedded in a traffic light system [47].

**Features of high-quality apps based on the Mobile App Rating Scale.**

- **General features:**
  1. Updates conducted in 2018 or 2019: recent updates to ensure that glitches are resolved
  2. Data security: developer ensures data security, in accordance with mobile app regulatory statement, for example, Health Insurance Portability and Accountability Act compliance
  3. Data exporting and sharing to clinicians: ability to link information readily to the clinician or the electronic health record
  4. Tracking of dental appointments: ability to record dental appointments
  5. Online social media support: ability to connect to social media networks such as Facebook

- **Features related to oral hygiene:**
  1. Tooth brushing timer: timer to encourage patients to brush for a certain amount of time 
  2. Tips for better oral hygiene: evidence-based information to improve oral hygiene
  3. Video demonstration: demonstration of brushing techniques via videos
  4. Goal setting: ability for user to input specific oral hygiene focused goal
  5. Tracking of oral health behavior: availability of statistics and charts on trends and adherence rates 
  6. Push notifications (reminders): alert on the phone to remind patients to behavior change, for example, brushing their teeth
  7. Gamification: apps that use game elements to encourage users to brush their teeth. This can include virtual reality battles to encourage brushing length
  8. Incentivization: earn prizes for virtual characters or cryptocurrency—may be reduce the cost of your next appointment 
  9. Sync to other apps on the phone: for example, the app may sync to a playlist to encourage brushing 

- **Features related to fluoride:**
  1. Provision of fluoride information: information about fluoride usage is in accordance with country guidelines
  2. Pictures of the amount of toothpaste: visual aids to demonstrate the amount of fluoride toothpaste that is to be placed on the toothbrush

- **Features related to diet:**
  1. Provision of dietary information: information about the connection between dietary habits and dental caries information on alternatives
  2. Diet diary: ability for the user to input food intake and time of consumption
  3. Text search for food items: search bar to allow ease of entry of the food item consumed
  4. Pictures of food: picture of food to correspond to diet diary with a traffic light grading system to encourage users to consider low sugar alternatives
  5. Serving size of food: ability to record the amount and not just the type of food consumed

### Quality Appraisal of Apps

Of the 40 apps, 18 (45%) were considered *high quality*, determined by reaching the minimum overall MARS threshold score of 3.0 out of 5.0. However, the MARS quality rating for each of the 40 apps found only 10 (25%) of these apps scored above 3.0 in all 4 subscales (Multimedia Appendix 4). The results also did not indicate that any single item in either of the 4 MARS subscales stood out. The interrater reliability between the reviewers, calculated from the overall and subscale scores of MARS for all apps, was excellent through the use of a two-way mixed intraclass correlation coefficient of 0.907 (95% CI 0.873-0.932). The *Brush DJ* app was the highest rated app with an overall MARS score of 4.4. This app also scored above the threshold for all subscales. The *Brush DJ* was also the only app that had scientific literature published, where a cross-sectional user acceptability questionnaire demonstrated 70.0% (133/189) of participants self-reported that the app motivated them to brush their teeth for longer [48]. However, the clinical effectiveness of this app is yet to be trialed using a study design that measures clinical health outcomes. Given the availability of only 1 app with supporting scientific literature published in this emerging field of inquiry, we followed the methodology adopted by other researchers in this situation [41] and excluded item 19 from our final calculation of the overall information subscale.

### Data Analysis and Synthesis

Further data analysis and synthesis was developed based on an iteratively generated hypothesis from the initial MARS quality analysis. First, we wanted to compare MARS scores between apps that addressed a differing number and range of prevention factors. Second, because the app that had the highest MARS rating also had the highest number of features, we hypothesized a correlation between MARS scores and the number of features identified in each app. Table 1 shows that apps addressing the oral hygiene factor had the highest mean overall MARS scores (3.3) compared with apps addressing a combination of other factors: diet (2.2), oral hygiene and fluoride (1.9), and oral hygiene and diet (2.2). Apps addressing oral hygiene alone also had the highest subscale scores in engagement (3.3), functionality (3.8), and aesthetics (3.3). Although oral hygiene apps ranked equal to apps that addressed all 3 factors in the mean information subscale, with a MARS score (2.9), the percentage of apps that were considered high quality was more consistent for apps addressing all 3 factors (8/15, 54%; Table 1). Apps that addressed the oral hygiene factor alone had the highest percentage of apps that were considered high quality in the engagement (10/17, 59%) and esthetic (13/17, 76%) subscales. Apps that addressed all 3 factors were more likely to score above the threshold in the MARS information subscale (8/15, 54%) compared with apps that addressed 1 (7/20, 35%) factor. Of the apps, 20% (3/15) that addressed all 3 factors also ranked comparatively poorly on engagement scores compared with 59% of apps (10/17) that addressed only the oral hygiene factor.

**Table 1:** Mobile App Rating Scale quality rating summary in dental caries prevention factor categories.

MARS^a^ subscale	1 factor				2 factors				3 factors	
	Oral hygiene only (n=17)		Diet only (n=3)		Oral hygiene and fluoride (n=2)		Oral hygiene and diet (n=3)		Oral hygiene, diet, and fluoride (n=15)	
	MARS score, mean (SD)	Quality apps^b^, n (%)	MARS score, mean (SD)	Quality apps, n (%)	MARS score, mean (SD)	Quality apps, n (%)	MARS score, mean (SD)	Quality apps, n (%)	MARS score, mean (SD)	Quality apps, n (%)
Overall	3.3 (0.5)	10 (59)	2.7 (0.6)	0 (0)	1.9 (0.2)	0 (0)	2.6 (1.2)	1 (33)	2.9 (0.8)	6 (40)
Engagement	3.0 (0.6)	10 (59)	2.3 (0.5)	0 (0)	1.3 (0.1)	0 (0)	2.8 (1.3)	1 (33)	2.5 (0.9)	3 (20)
Functionality	3.9 (0.7)	15 (88)	3.8 (0.0)	3 (100)	2.6 (0.2)	0 (0)	2.6 (1.3)	1 (33)	3.6 (0.7)	12 (80)
Aesthetics	3.3 (0.7)	13 (76)	2.4 (0.2)	0 (0)	1.8 (0.2)	0 (0)	2.4 (1.4)	1 (33)	2.6 (0.7)	5 (33)
Information^c^	2.9 (0.7)	7 (41)	2.2 (1.3)	1 (33)	1.9 (0.4)	0 (0)	2.2 (1.3)	1 (33)	2.9 (0.8)	8 (54)

^a^MARS: Mobile App Rating Scale.

^b^Percentage of apps determined to be of high quality, determined by an overall score that reached above the minimum threshold score of above 3.0 out of 5.0.

^c^Item 19 of the information subscale was excluded from the final calculation, as only 1 app supported the scientific literature published in this emerging field of inquiry, a similar methodology adopted by other researchers in this context [41].

**Figure 6:**
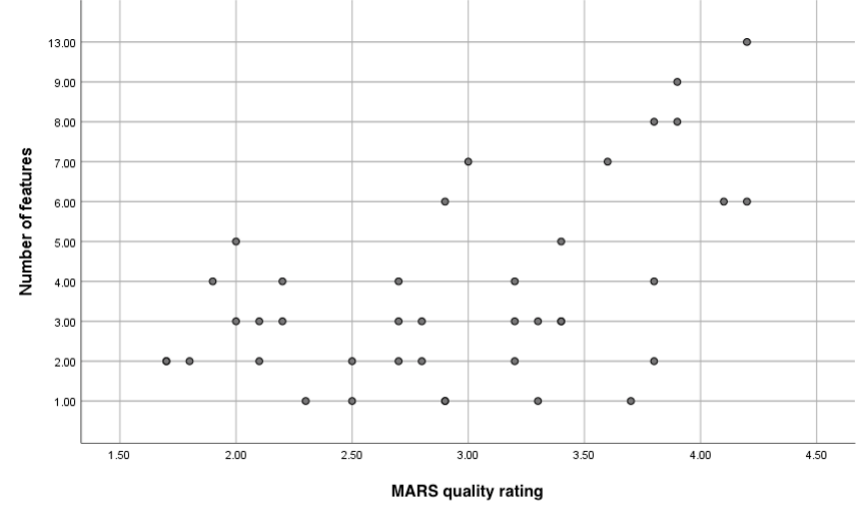
Number of features compared with the MARS quality rating scale for each app. MARS: Mobile App Rating Scale.

When analyzing the number of features, high-quality apps, categorized as those with an overall MARS score above 3, had almost double the mean number of features compared with low-quality apps, which was consistent across all categories (Multimedia Appendix 5). However, when comparing the individual apps with the MARS quality rating, the correlation between quality and the number of features showed variance and showed only a moderate general trend that high-quality apps had more features compared with low-quality apps (Figure 6).

## Discussion

### Principal Findings

Our study identified and assessed the characteristics, features, and quality of 40 apps targeted at the general public addressing a range of dental caries prevention factors. Less than half (18/40, 45%) of the apps were considered good quality (based on the overall MARS); however, only 25% (10/40) of apps were considered high quality across all 4 subscales of MARS. Most apps (37/40, 93%) focused on oral hygiene, and these apps were also more likely to contain a higher number of features and target the young adults or adolescent population. Only 13% (5/40) of apps addressed all 3 factors and were considered high quality, indicating only a small number of apps that could be further tested for clinical effectiveness specific to the adolescent or young adult populations. This could be recommended for the highest rated app, *Brush DJ*, which scored highly across all 4 subscales, demonstrating that it is possible to create an app that is able to provide good information and be aesthetically appealing. We did not find any high-quality apps targeted to parents of very young children that would address the specific dental caries prevention behaviors associated with caring for children aged between 0 and 6 years. Therefore, our study indicates an opportunity for future high-quality app development that addresses a range of dental caries prevention behaviors alongside a consideration for esthetics and engaging features to support this target parent population.

### Comparison With Prior Work

This study goes further than 2 previous reviews of mHealth apps focused on oral hygiene only [32] and oral health promotion in adult populations only [33] by scoping a broad range of dental caries prevention factors. Our study also responds to the need for feature analysis and quality appraisal outlined by the 2 most recent reviews on apps used for oral health [32,33]. First, our study provides the feature analysis of apps addressing a range of dental caries prevention behaviors, including diet modification and adequate fluoride usage. Second, the study provides an in-depth quality appraisal using the MARS tool. Consistent with the information quality concerns raised by these previous reviews, our analysis found 24 of 40 (60%) apps to be of low quality according to the MARS tool, yet still available to the general public. To address the issue of app quality [32], the National Health Service in the United Kingdom has created a digital app library that could be a trusted source of information for both clinicians and patients [49].

Our quality appraisal using the MARS assessment identified *Brush DJ* to be the highest-ranking app in quality and features, a similar finding from previously published reviews focused on oral hygiene in the United Kingdom [32]. At present, the *Brush DJ* app is the only app endorsed by the National Health Service’s digital app library [50]. Further well-designed clinical trials to determine the clinical efficacy of this app within the adolescent or young adult target populations should be undertaken. These clinical trials should have a clear clinical question with a good study design and be a randomized controlled trial where possible, with defined measurable health outcomes and a complementary economic evaluation [51]. Clinical effectiveness shown through improvements in measurable health outcomes will facilitate a more widespread adoption of this app and other effective apps in clinical practice.

### Implications

Dental caries is a multifactorial disease with varied risk factors that may impact individual patients differently during their life course [2,3]. Our study identified a lack of apps targeted to parents of children that adequately addressed prevention behaviors associated with fluoride usage and low-cariogenic diets for children aged younger than 6 years [3,52]. Given the paucity surrounding the clinical efficacy of mHealth apps in the field of dentistry [29], before a well-designed clinical trial can be conducted in this target population [48,53], a high-quality app needs to be designed. This app must target specific behaviors relevant to a broad range of dental caries prevention behaviors and contain evidence-based information, with appropriate features and esthetics to ensure the engagement of parents. To date, no high-quality app has achieved this. Thus, more user-focused, iterative co-designed research [54] relevant to the target population is needed to determine engaging features that will address all relevant dental caries prevention behaviors. Our assessment of features will provide a blueprint to assist future researchers engaged in qualitative user engagement research with parents, patients, clinicians, professional dental associations, health services, and research organizations [51,55] to develop a high-quality app that could then be trialed for clinical effectiveness.

### Limitations

Our review was conducted in Australia in 2019, and the included apps were limited to those available in Australian app stores at the specific time of the systematic search. Most apps included in this review have been developed in predominately English-speaking countries outside Australia, the United Kingdom, Canada, Asia, and the United States. We recognize that there may be apps developed in other languages or only available in country-specific app stores that were not included in our review. However, we did find a similar number of apps for the final analysis when using search terms similar to previous studies undertaken in other countries, including the United Kingdom [32] and the United States [33]. Second, in our study, MARS was used by researchers with clinical backgrounds in the field of oral health and primarily reflected this perspective. Our scoping study did not involve patients as participants and highlights the importance of conducting further complementary research that involves end users and giving voice to the patient’s perspective during the development of future apps and mHealth interventions [56].

### Conclusions

The increasing use of mHealth apps driven by increasing public use of mobile devices presents a call for dental researchers, health system managers, policy makers, and health professionals to engage with and provide more rigorous scientific recommendations around oral health apps. Our study provides a systematic and detailed analysis of the current availability, target audience, quality, and features of apps targeted toward dental caries. Quality was variable across the apps and mainly targeted the adolescent and adult populations. The most common features found in high-quality apps, such as gamification and goal setting, still focus on oral hygiene factors. It is unclear if these features can be used to address other dental caries prevention factors such as fluoride and diet modification. There was also an identified gap in apps available to support the target audience of parents of young children. There is a real need to co-design and create apps that address a broad range of modifiable risk factors associated with dental caries targeted at parents of children aged younger than 6 years. To ensure the highest quality in apps, the co-design process should include the clinician, researcher, and patient perspectives on evidence-based information and engaging features. Further studies are needed to determine the clinical efficacy of these apps before they can be widely recommended.

## Appendix

Multimedia Appendix 1 Mobile App Rating Scale tool developed by Queensland University of Technology (QUT).

Multimedia Appendix 2 General characteristics of all apps.

Multimedia Appendix 3 Comparative analysis of quality features present in all apps.

Multimedia Appendix 4 Quality evaluation of all apps according to the objective subscales of the Mobile App Rating Scale (MARS) quality appraisal tool.

Multimedia Appendix 5 Mean number of features present in high-quality versus low-quality apps within dental caries prevention factors.

## Abbreviations

MARS: Mobile App Rating Scale
mHealth: mobile health
