# Supplementary material for: Mobile Apps for Dental Caries Prevention: Systematic Search and Quality Evaluation
Source: JMIR Mhealth Uhealth. 2021 Jan 13;9(1):e19958. doi: 10.2196/19958 (PMC7840287; doi:10.2196/19958)
Supplement: Multimedia Appendix 2 [file mhealth_v9i1e19958_app2.docx]

Multimedia Table 1 General characteristics of all 40 included apps.

| **App name** | **Developer** | **Last Update** | **Platform** | **Star rating** | | **Affiliation ^a^** | **Target Age group^b^** | **Focus^c^** |
| --- | --- | --- | --- | --- | --- | --- | --- | --- |
|  | | | | **Google** | **iTunes** |  | | |
| 2mn Chrono - Brush your teeth | App Be Cool | 24/5/18 | iTunes^e^ | - | NE | COM | GEN | BC; GS |
| Baby Panda's Toothbrush | BabyBus Kids Games | 29/12/18 | Google | 4.2 | - | UNK | CHILD | BC |
| Best Toothbrushing Timer | UzmaDesign Studio | 13/4/17 | Google | 5 | - | UNK | GEN | BC; GS |
| Brush Teeth with The Wiggles | Australian Unity | 1/8/16 | Both | 4.3 | 4 | COM | CHILD | BC; GS |
| Brush Up | GamesThatWork | 25/7/18 | Google | 3.8 | - | COM; GOV; UNI | CHILD | BC; GS |
| Brushing Hero - Toothbrushing RPG | LITALICO Inc. | 12/12/18 | iTunes | 4.7 | - | UNK | CHILD | BC |
| Brush'n'save | AmanoDentalClinic | 28/2/17 | Google | 4.1 | - | COM | CHILD | BC; GS |
| BT's Dental Toothbrush Timer | InnovonX | 14/11/15 | Google | 3.6 | - | UNK | GEN | BC; GS |
| Dental Care - Target Smile | Kumar Veetrag | 10/4/16 | Google | 4.8 | - | UNK | YADL; ADL | BC; GS |
| Dental Desk | Dr.Prasad Joshi | 8/4/15 | Google | 4.6 | - | UNK | YADL; ADL | IO |
| Dental First Aid | The2thDr Apps | 15/10/17 | Google | 5 | - | COM | YADL; ADL | IO |
| Disney Magic Timer - by Oral B | Disney Publishing World Wide | 17/8/18 | Both | 3.9 | 4 | COM | CHILD | BC |
| Happy Kids Timer - Morning & Evening Chores | Kids Smart Zone | 14/12/18 | Google | 4.5 | - | UNK | CHILD | BC; GS |
| Toothbrush timer | Japps Medical | 30/9/16 | Google | 3.5 | - | UNK | GEN | BC; GS |
| Toothbrush Timer | QweQwe AppLabs | 2/8/14 | Google | 3.8 | - | UNK | GEN | BC; GS |
| TVOKids Tooth Time | TVO | 3/3/15 | Both | 3.4 | NE | COM | CHILD | BC; GS |
| WoodieHoo Brushing Teeth | RTL DISNEY Fernsehen GmbH & Co. KG | 26/11/18 | Both^e^ | 4 | NE | COM | CHILD | BC |
| How to Heal Cavities Naturally | StatesApps | 7/1/18 | Google | 2.3 | - | UNK | ADOL; YADL; ADL | IO |
| Toothache | Jackline moline | 20/8/18 | Google | NE | - | UNK | YADL; ADL | IO |
| My Teeth - Cleaner teeth while having fun | Vogelbusch & Co | 1/1/19 | iTunes^e^ | - | NE | COM | CHILD | BC; GS |
| Tooth Decay | Everyone Learning App | 11/10/18 | Google | 5 | - | UNK | ADOL; YADL; ADL | IO |
| TOOTHACHE REMEDY TIPS | Lizdin Enterprise | 7/2/18 | Google | 4.2 | - | UNK | ADOL; YADL; ADL | IO |
| Cavity | EveryoneLearning Apps | 26/9/18 | Google | 3 | - | UNK | ADOL; YADL; ADL | IO |
| FoodForTeeth- Food Database and Diet Diary | Prateek Biyani | 17/5/17 | iTunes | - | NE | UNK | ADOL; YADL; ADL | BC |
| Tooth Decay Advice | moreFlow | 7/4/18 | Google | 5 | - | UNK | YADL; ADL | IO |
| All Dental Disorders | Mynarh_Apps | 23/11/18 | Google | 5 | - | UNK | YADL; ADL | IO |
| Brush DJ | Ben Underwood | 25/9/18 | Both | 4.1 | 4.7 | GOV; UNI | GEN | BC; GS |
| Dental Care | MSPLDevelopers | 29/9/18 | Google | 4.6 | - | UNK | ADOL; YADL; ADL | IO |
| Dental Care Tips | adamsumar | 27/9/18 | Google | 5 | - | UNK | ADOL; YADL; ADL | IO |
| Dentist G | Gaurav dixit | 27/3/17 | Google | 4.9 | - | UNK | ADOL; YADL; ADL | IO |
| DRINKS DESTROY TEETH | Indiana Dental Association | 16/6/15 | Both | 4.6 | NE | NGO; UNI | ADOL; YADL; ADL | BC; GS |
| How to Prevent Cavities | NonitaDev | 28/6/18 | Google | 5 | - | UNK | GEN | IO |
| How To Stop a Toothache | The Almighty Dollar | 26/7/18 | Google | NE | - | UNK | ADOL; YADL; ADL | IO |
| My Bright Smile | Colgate-Palmolive Company | 18/5/17 | Both | 3.8 | 5 | COM | CHILD | BC |
| My Dental-Care - Your Guide to Oral Health | Digiryte | 12/4/19 | Both | 5 | NE | GOV; UNI | ADOL; YADL; ADL | IO |
| Teeth Care | dagana Apps | 28/9/18 | Google | 5 | - | UNK | ADOL; YADL; ADL | IO |
| tooth decay | Digital Planete Space | 1/6/18 | Google | 4.5 | - | UNK | ADOL; YADL; ADL | IO |
| Toothache | Digital Planete Space | 1/6/18 | Google | NE | - | UNK | YADL; ADL | IO |
| Toothache: Causes, Diagnosis, and Management | Health Info | 15/9/18 | Google | 5 | - | UNK | ADOL; YADL; ADL | IO |
| WhenToDoctor - Symptom Checker & Medical Advice | Esma Universalis | 21/9/18 | Google | 3.6 | - | UNK | YADL; ADL | IO |

Legend

^a^ Affiliations of the creation of the App: UNK: unknown, COM: Commercial; GOV: Government; NGO: Non-government organisation; UNI: University.

^b^ Target age group of the App: CHILD: Children under 12, ADOL: Adolescents 13-17, YADUL: Young Adults between 18-25, ADUL: Adults, GEN: General audience.

^c^ Focus of the App : Focus of the BC: Behaviour Change, GS: Goals Setting, IO: Information Only

^d^ NE: Not enough downloads or reviews to generate an average star rating on this platform.

^e^ Apps that cost between 0.99-2.99AUD to download
