# Supplementary material for: Mobile Apps for Dental Caries Prevention: Systematic Search and Quality Evaluation
Source: JMIR Mhealth Uhealth. 2021 Jan 13;9(1):e19958. doi: 10.2196/19958 (PMC7840287; doi:10.2196/19958)
Supplement: Multimedia Appendix 3 [file mhealth_v9i1e19958_app3.docx]

Comparative identification of quality features present in all apps.

| App name | General features | | | | | Oral hygiene features | | | | | | | | | Fluoride features | | Diet features | | | | |
| --- | --- | --- | --- | --- | --- | --- | --- | --- | --- | --- | --- | --- | --- | --- | --- | --- | --- | --- | --- | --- | --- |
| **Features ^a^ corresponding to Table2** | **1** | **2** | **3** | **4** | **5** | **6** | **7** | **8** | **9** | **10** | **11** | **12** | **13** | **14** | **15** | **16** | **17** | **18** | **19** | **20** | **21** |
| **Oral Hygiene only** | | | | | | | | | | | | | | | | | | | | |  |
| 2mn Chrono - Brush your teeth | ✓ |  | - | - | - | ✓ | - | - | ✓ | - | - | - | - | - |  |  |  |  |  |  |  |
| Baby Panda's Toothbrush | ✓ |  | - | - | - | - | - | - | - | - | - | ✓ | - | - |  |  |  |  |  |  |  |
| Best Toothbrushing Timer | - |  | - | - | - | ✓ | - | - | - | - | - | - | - | - |  |  |  |  |  |  |  |
| Brush Teeth with The Wiggles | - | ✓ | - | - | - | ✓ | ✓ | - | ✓ | ✓ | - | - | - | - |  |  |  |  |  |  |  |
| Brush Up | ✓ |  | - | - | - | ✓ | ✓ | - | ✓ | - | ✓ | ✓ | - | - |  |  |  |  |  |  |  |
| Brushing Hero - Toothbrushing RPG | ✓ |  | - | - | - | ✓ | - | - | - | - | ✓ | ✓ | - | - |  |  |  |  |  |  |  |
| Brush'n'save | - |  | - | - | ✓ | ✓ | ✓ | ✓ | ✓ | ✓ | - | - | ✓ | - |  |  |  |  |  |  |  |
| BT's Dental Toothbrush Timer | - |  | - | - | - | ✓ | - | - | - | - | - | - | - | - |  |  |  |  |  |  |  |
| Dental Care - Target Smile | - |  | - | - | - | ✓ | - | - | - | ✓ | - | - | - | - |  |  |  |  |  |  |  |
| Dental Desk | - |  | - | - | - | ✓ | - | - | - | - | - | - | - | - |  |  |  |  |  |  |  |
| Dental First Aid | - |  | - | - | - | ✓ | - | - | - | - | - | - | - | - |  |  |  |  |  |  |  |
| Disney Magic Timer - by Oral B | - | ✓ | - | ✓ | ✓ | ✓ | ✓ | ✓ | ✓ | ✓ | ✓ | ✓ | - | - |  |  |  |  |  |  |  |
| Happy Kids Timer - Morning & Evening Chores | ✓ |  | - | - | - | ✓ | - | ✓ | ✓ | - | - | ✓ | - | - |  |  |  |  |  |  |  |
| Toothbrush timer | - |  | - | - | - | ✓ | - | - | - | - | - | - | - | - |  |  |  |  |  |  |  |
| Toothbrush Timer | - |  | - | - | - | ✓ | - | - | - | - | - | - | - | - |  |  |  |  |  |  |  |
| TVOKids Tooth Time | - |  | - | - | - | ✓ | - | - | ✓ | - | - | - | - | - |  |  |  |  |  |  |  |
| WoodieHoo Brushing Teeth | ✓ |  |  | - | - | ✓ | ✓ | - | - | - | - | ✓ | - | - |  |  |  |  |  |  |  |
| **Oral Hygiene and Fluoride** | | | | | | | | | | | | | | | | | | | | |  |
| How to Heal Cavities Naturally | ✓ |  | - | - | - | - | - | - | - | - | - | - | - | - | ✓ | - |  |  |  |  |  |
| Toothache | ✓ |  | - | - | - | - | - | - | - | - | - | - | - | - | ✓ | - |  |  |  |  |  |
| **Oral Hygiene and Diet** | | | | | | | | | | | | | | | | | | | | |  |
| My Teeth - Cleaner teeth while having fun | ✓ |  | - | - | - | ✓ | ✓ | ✓ | ✓ | - | - | ✓ | - | ✓ |  |  | ✓ | - | - | - | - |
| Tooth Decay | ✓ |  | - | - | ✓ | - | ✓ | - | - | - | - | - | - | - |  |  | ✓ | - | - | - | - |
| TOOTHACHE REMEDY TIPS | ✓ |  | - | - | - | - | - | - | - | - | - | - | - | - |  |  | ✓ | - | - | - | - |
| **Diet only** | | | | | | | | | | | | | | | | | | | | |  |
| Cavity | ✓ |  | - | - | ✓ |  |  |  |  |  |  |  |  |  |  |  | ✓ | - | - | - | - |
| DRINKS DESTROY TEETH | ✓ |  | - | - | ✓ |  |  |  |  |  |  |  |  |  |  |  | ✓ | - | - | - | - |
| Tooth Decay Advice | ✓ |  | - | - | - |  |  |  |  |  |  |  |  |  |  |  | ✓ | - | - | - | - |
| **Oral Hygiene, Fluoride and Diet** | | | | | | | | | | | | | | | | | | | | |  |
| All Dental Disorders | ✓ |  | - | - | - | - | - | - | - | - | - | - | - | - | ✓ | - | ✓ | - | - | - | - |
| Brush DJ | - | ✓ | - | ✓ | ✓ | ✓ | ✓ | ✓ | ✓ | ✓ | ✓ | - | - | ✓ | ✓ | ✓ | ✓ | - | - | - | - |
| Dental Care | ✓ |  | - | - | - | - | - | - | - | - | - | - | - | - | ✓ | - | ✓ | - | - | - | - |
| Dental Care Tips | - |  | - | - | - | - | - | - | - | - | - | - | - | - | ✓ | - | ✓ | - | - | - | - |
| Dentist G | - |  | - | - | - | - | - | - | - | - | - | - | - | - | ✓ | - | ✓ | - | - | - | - |
| FoodForTeeth- Food Database and Diet Diary | - |  | ✓ | - | ✓ | - | - | - | - | - | - | - | - | - | ✓ | - | ✓ | ✓ | ✓ | ✓ | ✓ |
| How to Prevent Cavities | ✓ |  | - | - | ✓ | - | - | - | - | - | - | - | - | - | ✓ | - | ✓ | - | - | - | - |
| How To Stop a Toothache | ✓ |  | - | - | ✓ | - | - | - | - | - | - | - | - | - | ✓ | - | ✓ | - | - | - | - |
| My Bright Smile | - | ✓ | - | - | ✓ | ✓ | - | - | - | - | - | ✓ | - | - | ✓ | - | ✓ | - | - | - | - |
| My Dental-Care - Your Guide to Oral Health | ✓ | ✓ | - | - | ✓ | - | ✓ | - | - | - | - | - | - | - | ✓ | ✓ | ✓ | - | - | - | - |
| Teeth Care | ✓ |  | - | - | - | - | - | - | - | - | - | - | - | - | ✓ | - | ✓ | - | - | - | - |
| tooth decay | ✓ |  | - | - | ✓ | - | ✓ | - | - | - | - | - | - | - | ✓ | - | ✓ | - | - | - | - |
| Toothache | ✓ |  | - | - | - | - | - | - | - | - | - | - | - | - | ✓ | - | ✓ | - | - | - | - |
| Toothache: Causes, Diagnosis, and Management | ✓ |  | - | - | - | - | - | - | - | - | - | - | - | - | ✓ | - | ✓ | - | - | - | - |
| WhenToDoctor - Symptom Checker & Medical Advice | ✓ | - | - | - | - | - | - | - | - | - | - | - | - | - | ✓ | - | ✓ | - | - | - | - |

Legend

✓ Feature is present in the app

^a^ Features are in correspondence to the comprehensive list of features in Table 2
