# Supplementary material for: Mobile Apps for Dental Caries Prevention: Systematic Search and Quality Evaluation
Source: JMIR Mhealth Uhealth. 2021 Jan 13;9(1):e19958. doi: 10.2196/19958 (PMC7840287; doi:10.2196/19958)
Supplement: Multimedia Appendix 4 [file mhealth_v9i1e19958_app4.docx]

Quality evaluation of all apps according to the objective subscales of the MARS^a^ quality appraisal tool.

| App name | Engagement score | Functionality score | Aesthetic score | Information score^b^ | Overall MARS score |
| --- | --- | --- | --- | --- | --- |
| **Oral hygiene only** | | | | | |
| 2mn Chrono - Brush your teeth | 3.0 | 4.5 | 3.4 | 3.2 | 3.5^d^ |
| Baby Panda's Toothbrush | 3.2 | 4.3 | 3.7 | 2.3 | 3.4^c^ |
| Best Toothbrushing Timer | 2.4 | 4.8 | 2.0 | 2.5 | 2.9 |
| Brush Teeth with The Wiggles | 3.0 | 4.5 | 3.7 | 2.8 | 3.5^c^ |
| Brush Up | 4.4 | 4.0 | 4.0 | 4.3 | 4.2^d^ |
| Brushing Hero - Toothbrushing RPG | 4.0 | 4.0 | 3.3 | 3.8 | 3.8^d^ |
| Brush'n'save | 2.6 | 3.8 | 3.0 | 2.5 | 3.0^c^ |
| BT's Dental Toothbrush Timer | 2.8 | 2.3 | 2.7 | 1.7 | 2.4 |
| Dental Care - Target Smile | 2.2 | 3.5 | 2.7 | 2.7 | 2.8 |
| Dental Desk | 2.4 | 2.5 | 2.3 | 2.7 | 2.5 |
| Dental First Aid | 3.4 | 4.0 | 4.0 | 3.5 | 3.7^d^ |
| Disney Magic Timer - by Oral B | 3.2 | 4.5 | 4.3 | 3.8 | 4.0^d^ |
| Happy Kids Timer - Morning & Evening Chores | 2.6 | 4.0 | 3.0 | 2.5 | 3.0^c^ |
| Toothbrush timer | 2.6 | 4.0 | 3.0 | 2.0 | 2.9 |
| Toothbrush Timer | 3.0 | 4.0 | 3.3 | 3.0 | 3.3^c^ |
| TVOKids Tooth Time | 3.4 | 4.3 | 4.3 | 3.5 | 3.9^d^ |
| WoodieHoo Brushing Teeth | 3.4 | 4.0 | 3.7 | 2.2 | 3.3^c^ |
| Mean | 3.0 | 3.9 | 3.3 | 2.9 | 3.3^c^ |
| **Oral Hygiene and Fluoride** | | | | | |
| How to Heal Cavities Naturally | 1.2 | 2.5 | 1.7 | 1.7 | 1.8 |
| Toothache | 1.4 | 2.8 | 2.0 | 2.2 | 2.1 |
| Mean | 1.3 | 2.7 | 1.9 | 2.0 | 2.0 |
| **Oral Hygiene and Diet** | | | | | |
| MyTeeth-Cleaner teeth havingfun | 4.2 | 3.8 | 4.0 | 3.7 | 3.9^d^ |
| Tooth Decay | 1.6 | 2.8 | 2.0 | 1.5 | 2.0 |
| TOOTHACHE REMEDY TIPS | 2.6 | 2.0 | 2.3 | 1.5 | 2.1 |
| Mean | 2.8 | 2.9 | 2.8 | 2.2 | 2.7 |
| **Diet only** | | | | | |
| Cavity | 1.8 | 3.8 | 2.3 | 1.2 | 2.3 |
| DRINKS DESTROY TEETH | 2.8 | 3.8 | 2.7 | 3.7 | 3.3^c^ |
| Tooth Decay Advice | 2.4 | 3.8 | 2.3 | 1.8 | 2.6 |
| Mean | 2.3 | 3.8 | 2.4 | 2.2 | 2.7 |
| **Diet, Fluoride and Oral Hygiene** | | | | | |
| All Dental Disorders | 2.6 | 4.8 | 3.3 | 3.2 | 3.5^c^ |
| Brush DJ | 4.2 | 4.3 | 4.3 | 4.7 | 4.4^d^ |
| Dental Care | 2.2 | 3.8 | 2.3 | 2.8 | 2.8 |
| Dental Care Tips | 1.8 | 2.3 | 1.7 | 1.7 | 1.9 |
| Dentist G | 2.4 | 3.5 | 2.3 | 3.0 | 2.8 |
| Food For Teeth- Food Database and Diet Diary | 3.8 | 3.8 | 3.3 | 4.2 | 3.8^d^ |
| How to Prevent Cavities | 1.8 | 3.8 | 2.3 | 3.0 | 2.7 |
| How To Stop a Toothache | 1.8 | 3.3 | 2.0 | 2.0 | 2.3 |
| My Bright Smile | 4.2 | 4.3 | 4.0 | 3.8 | 4.1^d^ |
| My Dental-Care - Your Guide to Oral Health | 2.8 | 3.0 | 3.0 | 4.2 | 3.3^c^ |
| Teeth Care | 2.4 | 3.5 | 2.7 | 2.5 | 2.8 |
| tooth decay | 1.8 | 2.8 | 2.3 | 1.5 | 2.1 |
| Toothache | 1.4 | 2.8 | 2.0 | 2.0 | 2.1 |
| Toothache: Causes, Diagnosis, and Management | 2.2 | 3.3 | 1.7 | 1.7 | 2.2 |
| WhenToDoctor-Symptom Checker & Medical Advice | 1.8 | 4.0 | 1.7 | 3.6 | 2.8 |
| Mean | 2.5 | 3.6 | 2.6 | 2.9 | 2.9 |
| Mean of all 40 apps | 2.7 | 3.6 | 2.9 | 2.8 | 2.9 |

^a^ The Mobile Application Rating Scale MARS is a quality rating scale that appraises the quality of an app using a 5-point scale across four subscales including: engagement, functionality, aesthetics, and information.

^b^ Item 19 of the Information subscale was excluded from the final calculation as only one app has supporting scientific literature published in this emerging field of enquiry, a similar methodology adopted by other researchers in this context [41].

^c^ Apps with an overall score that reached the minimum threshold score of above 3.0

^d^ Apps that reached the minimum threshold score of above 3.0 in the overall scale and across all four other subscales
