# Supplementary material for: Mobile Apps for Dental Caries Prevention: Systematic Search and Quality Evaluation
Source: JMIR Mhealth Uhealth. 2021 Jan 13;9(1):e19958. doi: 10.2196/19958 (PMC7840287; doi:10.2196/19958)
Supplement: Multimedia Appendix 5 [file mhealth_v9i1e19958_app5.docx]

Mean number of features present in high quality vs. low quality apps categorised within prevention categories.

| **Mean number of features present in high quality vs. low quality apps within prevention categories** | | | | | | |
| --- | --- | --- | --- | --- | --- | --- |
|  | Oral hygiene | | Fluoride | | Diet | |
|  | High Quality | Low Quality | High Quality | Low Quality | High Quality | Low Quality |
| Number of apps in the category | 17 | 20 | 5 | 12 | 7 | 14 |
| Mean number of features | 5.24 | 2.70 | 7.40 | 3.00 | 6.86 | 3.07 |
| Std. Deviation | 3.231 | 1.380 | 3.647 | .953 | 3.436 | .917 |
| Variance | 10.441 | 1.905 | 13.300 | .909 | 11.810 | .841 |
| Range | 12 | 5 | 10 | 3 | 10 | 3 |
| Minimum | 1 | 1 | 3 | 2 | 3 | 2 |
| Maximum | 13 | 6 | 13 | 5 | 13 | 5 |
